# Sedentary and Physical Activity Patterns in Adults with Intellectual Disability

**DOI:** 10.3390/ijerph14091027

**Published:** 2017-09-07

**Authors:** Guillermo R. Oviedo, Noémie Travier, Myriam Guerra-Balic

**Affiliations:** 1 FPCEE-Blanquerna, University Ramon Llull, 34 Císter Street, 08022 Barcelona, Spain; miriamelisagb@blanquerna.url.edu; 2 Physical Activity, Sport and Recreation Research Focus Area, Faculty of Health Sciences, North-West University, Potchefstroom 2520, South Africa; 3 Unit of Nutrition and Cancer, Catalan Institute of Oncology (ICO-IDIBELL), L’Hospitalet de Llobregat, 08907 Barcelona, Spain; ntravier@iconcologia.net

**Keywords:** intellectual disability, physical activity, sedentarism, ageing, accelerometer

## Abstract

Little is known about the patterns of sedentary time (ST) and physical activity (PA) levels throughout the week among adults and older adults with Intellectual Disability (ID). We analyzed ST and PA patterns of adults and older adults with ID. Forty-two adults and 42 older adults with mild to severe ID participated in this study. Height and weight were obtained to calculate Body Mass Index (BMI). Body fat and fat-free mass percentages were also obtained. Patterns of PA levels and ST were assessed with GT3X Actigraph accelerometers. Adults performed higher amounts of total PA and moderate to vigorous PA than older adults during the week, on weekdays and in center time (all *p* > 0.05). No differences between males and females were found for either PA levels or ST. Only 10.7% of the participants met the global recommendations on PA for health. The participants of the current study showed low PA levels and a high prevalence of ST. Interestingly, when comparing age and/or sex groups, no differences were observed for ST. Our findings provide novel and valuable information to be considered in future interventions aiming to increase PA levels and reduce ST.

## 1. Introduction

The lack of physical activity (PA) and the increase of sedentary behaviours among the general population have shown a direct relation with negative health outcomes [1,2]. For this reason many health guidelines published by several health authorities recommend regular PA to prevent health impairments [3,4].

There is a need to better understand PA levels, its nature and its relation to several health indicators. That is why researchers create and improve the tools used to quantify PA levels in different populations [5,6]. As previous studies have already shown over the few last years, numerous instruments have been developed to objectively measure PA levels. Accelerometers are used to objectively monitor PA, and lately, their use in different populations has increased [6,7]. There are studies that have effectively used these motion sensors with participants with intellectual disability (ID) to assess sedentary time (ST), PA levels and energy expenditure [8,9,10,11].

The Sedentary Behaviour Research Network has defined sedentary behaviour as activities with energy expenditures ≤1.5 metabolic equivalents (MET) while in a sitting or reclining posture during waking hours [12]. This behaviour is related to negative effects on health [13,14]. To counteract these effects, adults should get at least 150 min a week of moderate PA (MPA), which intensities are between 3 to 5.99 METs, or 75 min a week of vigorous PA (VPA) that correspond to intensities ≥6.00 METs in order to obtain metabolic health benefits [15]. These activities should be in bouts of at least 10 min [4]. As specific PA guidelines for people with ID do not exist, the general population recommendations are the guidelines used. More recently, awareness has risen about the importance of breaking-up ST and sedentary bouts as it was found that breaking-up ST and decreasing sedentary bouts are independent factors related to health outcomes and activities of daily living [16,17,18].

Regular PA is essential for adults with ID [19] because they show low rates of PA, decreased cardiovascular fitness and, high incidence of obesity [20,21,22]. Also, people with ID suffer from an early aging process starting around age 40–50 [23,24]. This process causes an early appearance of physical health problems, musculoskeletal disability, visual and hearing problems, dementia, chronic illnesses, and can, therefore, affect the activities of daily living [25,26]. In Spain, adults with ID older than 45 years old are considered older adults because the frequency of suffering these illnesses are similar to people older than 65 years old without ID [27].

Emerging evidence suggests that adults with ID are less active than healthy adults without ID [11,28]. In their review, Temple et al. [22] concluded that almost two-thirds of adults with ID did not reach the minimum amount of PA that might provide health benefits. Other studies showed that approximately 17% to 21% of adults and older adults with ID achieved the recommended 10,000 steps per day [29,30]. Furthermore, different authors have shown that most of the waking time during the day was spent being sedentary [21,31,32]. The study by Dixon-Ibarra et al. [33] indicated that older adults with ID were less active than a younger group with ID and a group of older adults without ID. In addition, they reported that 6% of older adults with ID and 13% of younger adults with ID met the recommendations of moderate to vigorous PA (MVPA) and ST ranged from 60% to 65% of waking time.

Little is known about the actual patterns of ST, as well as the duration and breaks of this behaviour, and different studies [11,28] pointed out the necessity of further studies to better understand sedentary behaviours and PA levels in people with ID. Due to the increasing lifespan of people with ID [34,35], it is important to know the bout and patterns of PA and ST of adults and older adults with ID, because such data could be used to design and implement age-specific health promotion programs aimed at decreasing sedentariness and increasing PA levels.

As far as we know, there are no studies that examined the temporal patterning of ST and PA levels throughout the week in adults and older adults with ID. Therefore, the purpose of this study was to objectively assess the temporal patterning of ST and PA levels throughout the week of Spanish adults and older adults with ID using accelerometers. A secondary purpose was to analyze the potential age and sex differences in PA levels and ST patterns among adults and older adults with ID throughout the week.

## 2. Materials and Methods

### 2.1. Study Design and Participants

This cross-sectional study used a convenience sample from an Occupational Day Center for people with ID (Catalonia, Spain). At the Occupational Day center, participants perform activities like sewing cushion covers and rugs, arts and crafts, weaving baskets, computer work, watching TV or videos, reading, writing, cleaning and, gardening. A total of 60 adults <45 years old (32 males/28 females) and 56 older adults ≥45 years old (35 males/21 females), with mild to severe ID from the center, were invited to participate in this study. The informed consent form to participate in this study was obtained from 40 women and 52 men (45 ± 12 years old; 79.3% of the invited participants) with mild (*n* = 30), moderate (*n* = 34) and severe ID (*n* = 28), and their parents/legal tutors as requested by the Spanish law.

After signing the informed consent form, the ID classification was obtained from the patients’ medical records resulting that all the participants were diagnosed with mild to severe ID according to the Spanish National Government classification [36]. This classification of ID represents a combination of patients’ level of intelligence quotient (IQ) and adaptive behaviour, and categorizes the percentage of disability (physical, intellectual and/or sensorial) in 5 degrees as follows: non-existent (0%), border-line (15–29%), mild (30–59%), moderate (60–75%)and severe or very severe (≥76%).

All participants presented ID, but in 72 participants we were not able to classify the ID etiology. The other 20 were diagnosed as Down syndrome (*n* = 13), West syndrome (*n* = 2), cerebral palsy (*n* = 2), Cornelia Lange syndrome (*n* = 2) and Microcephaly (*n* = 1). Among these 20 participants conduct disorder (*n* = 13); epilepsy (*n* = 3) and autism (*n* = 2) were also diagnosed.

### 2.2. Procedures

Before beginning the study two meetings were held with the participants and parents/legal tutors. During the first meeting, testing procedures, potential benefits, associated risks and the period of time required for the study were explained to the participants and parents/legal tutors. During the second meeting, the different assessments were carried out. A rigorous process and procedure of obtaining consent for the study was set out to allow participants to be fully informed, autonomous, and empowered to consent as part of the study. Participants and parents and/or legal guardians were provided with space and time to assent to participate in the study. All participants and parents and/or legal guardians signed an informed consent to participate. After signing the informed consent form, a health screening questionnaire was completed by each participant’s parent(s) and/or guardian.

All participants were able to walk without aids and did not present motor impairments. Exclusion criteria included contraindications to exercise or presence of a physical disability that would not allow doing PA.

This study was approved by the Institutional Review Board of the University Ramon Llull and follows the Helsinki guidelines for ethical behaviour (Ethical code: 1112004P).

### 2.3. Anthropometric Measures

Height was measured to the nearest 0.1 cm by using a stadiometer (Seca 225, Seca, Hamburg, Germany). Weight was measured to the nearest 0.1 kg on a digital scale (Seca 861, Hamburg, Germany) with the subject wearing lightweight clothing and no shoes. Body mass index (BMI) was calculated as weight in kilograms divided by height in meters squared (kg/m^2^) and participants were categorised into normal weight (BMI < 25), overweight (BMI of 25–29.99), and obese (BMI ≥ 30) [37]. Waist circumference (WC) was measured three times in a horizontal plane at the narrowest section between the hips and the bottom of the rib cage. Participants were categorised into normal or elevated WC (WC </≥ 88 cm in women and WC </≥ 102 cm in men) [37]. Chest, abdomen and thigh skinfolds thickness for men and triceps, suprailiac, and thigh skinfolds thickness for women were measured on the right side of the body by using a Holtain skinfold caliper (Holtain Ltd., Wales, UK) and following the International Society for the Advancement of Kinanthropometry guidelines [38]. To calculate body density, the equation of Jackson and Pollock [39] was used for men. For women the equation of Jackson, Pollock and Ward [40] was used. Body fat percentage (BF%) was calculated using Siri’s equation [41] and body fat-free mass percentage (FFM%) was determined thereafter [42]. All measurements were taken three times by the same trained person and the mean of the two closest was used for the analysis (test-retest reliability score *r* = 0.95).

### 2.4. Physical Activity Measurement

Physical activity was assessed by using the GT3X ActiGraph accelerometer (Firmware 4.4.0, ActiGraph™, Fort Walton Beach, FL, USA) and data were downloaded with the ActiLife 6 Software (v.6.12.0., ActiGraph™, Fort Walton Beach, FL, USA). This model, released in 2009, detects steps differently than the AM7164 model, therefore, applying the process of censoring steps, may no longer be necessary if a pedometer-based comparison is required [43,44,45].

Participants and parents/legal guardians were provided with instructions on how to wear the accelerometer during all waking hours, its placement and wear time. The accelerometers were fitted to the participants’ right hip and were collected 8 days later. The devices were not used while bathing, showering or swimming.

Outcome variables were total physical activity (TPA) (counts·min^−1^), steps per day (steps·day^−1^), and time spent (min·day^−1^) in sedentary (ST) (0 to 99 counts·min^−1^), light (LPA) (100 to 2019 counts·min^−1^) and MVPA (≥2020 counts·min^−1^). Time in MVPA was determined by adding the minutes over the moderate intensity threshold. The intensity cutoff points were replicated from the National Health and Nutritional Examination Survey (NHANES) for adults over 18 years of age [46]. At the same time, accelerometer data were divided into week days (WD) (Monday–Friday) and weekend days (WeD) (Saturday and Sunday), and in center time (9 a.m. to 5 p.m.) and outside-center time (from 5 p.m. to 11:59 p.m.) on week days. Non-wearing time was defined as a period of 60 consecutive minutes of zero counts with an allowance for up to 2 min of counts between 1 and 100.

The proportion of participants meeting WHO recommendations for PA was assessed by following the methodology described by Troiano et al. (2008) [46]. We have also organized steps per day within 4 categories [47]: (1) ˂5000; (2) 5000–7499; (3) 7500–9999; (4) 10,000–12,499; and (5) ≥12,500.

Three further summary measures of ST were calculated per day and averaged over valid days: percentage of wear-day spent in ST; number of sedentary bouts (defined as a period of consecutive minutes where the accelerometer registers <100 counts·min^−1^) and number of sedentary breaks (defined as at least 1 min where the accelerometer registers ≥100 counts·min^−1^ following a sedentary bout).

The necessary number of days determined through the literature to assess habitual PA is four (week and/or weekend) days [48,49,50]. Participants who did not meet these criteria were given a new period to wear the monitors. For inclusion in the analysis, each participant needed a minimum wear time of 10 h per day [46]. Eight participants (3 older adults; 5 adults) that correspond to the 8.7% of the recruited participants did not meet the accelerometer criteria to be included in the analysis.

### 2.5. Statistical Analysis

Descriptive statistics were obtained for all variables, including sex, age, weight, height, BMI, WC, body fat and fat-free mass % and PA outcomes. Continuous variables are presented as means and standard deviation (SD), and categorical variables are presented as percentages. Chi-square tests were used to investigate differences in proportions between sex and age groups, while *t*-tests were used to compare means.

Linear mixed-effect models were used to evaluate the effect of age (adults vs. older adults), sex, time of the day (center time vs. outside-center time) and day of the week (WD vs. WeD) on ST and PA levels. The four variables age, sex, time of the day and day of the week were simultaneously introduced into the models as cofactors, as the log likelihood ratio tests previously performed to assess the possible interaction between age and sex were only found significant for MVPA. For this specific outcome, a stratified analysis was also performed. Linear mixed-effects models were used to address the facts that we had repeated measures (center time vs. outside-center time and week day vs. weekend) and unbalanced data. Indeed, since we used mixed models we did not have to exclude from the analysis the few individuals for whom the outcome variable was not available at one time point [51]. Finally, adjusted linear models were used to analyze differences in ST between sex and age groups, and BMI category.

All models were controlled for the differences in wear time during each period and all outcome variables were naturally log-transformed (Ln) before being used in the models. The critical values for statistical significance were assumed at an alpha level ≤0.05. Statistical analyses were conducted by using the Statistical Package for the Social Sciences (IBM SPSS, v 22.0, Chicago, IL, USA).

## 3. Results

### 3.1. Demographics and Descriptive Data

A total of 84 participants (49 males and 35 females) were included in the analysis. The general characteristics of the participants are presented in Table 1.

The proportion of participants categorized as obese was higher in females than males (*p* < 0.001). Regarding WC mean values, no differences were found for sex. However, older adults presented significantly higher WC mean values than adults (*p* < 0.001). The proportion of females categorized as elevated WC was higher than males (*p* < 0.001). Males had significantly lower BF% mean values and higher FFM% mean values than females (all *p* < 0.001).

### 3.2. Physical Activity and Sedentary Time Differences

Sedentary time and PA outcomes throughout the week are shown in Table 2. Participants wore the activity monitor during 5.99 ± 1.07 days, of which 4.38 ± 0.37 days during WD and 1.61 ± 0.60 days during WeD. During accelerometer wearing time, participants spent more time in ST during WD than during WeD, as well as during center time than during the outside-center time (all *p* < 0.001). Participants performed more LPA, MVPA, TPA and steps per day during WD than during WeD (all *p* < 0.001). When comparing center time vs. outside-center time, participants engaged more LPA, MVPA and steps per day (all *p* < 0.050) when they were at the center.

Table 3 depicts PA outcomes during WD, WeD, center time and outside-center time by sex and age group. A main effect was detected for age group, where adults performed more MVPA and TPA during all week (WD + WeD) than older adults (*p* = 0.011; *p* = 0.038). Adults performed more MVPA and TPA than older adults during WD (*p* = 0.014 and *p* = 0.042 respectively). Also, during center time, adults performed more MVPA and TPA and had a higher step count than older adults (all *p* < 0.050). However, the differences in MVPA and TPA observed between adults and older adults with ID during WD were mainly due to the significant differences observed in men (*p* = 0.0004.; *p* = 0.013 respectively). The differences in MVPA and TPA during center time were also only observed among men (*p* = 0.003; *p* = 0.019 respectively) (results not shown).

When analyzing differences between WD vs. WeD, it was found that all participants were more sedentary during WD (all *p* < 0.010). Regarding LPA and steps, all participants presented higher mean values during WD than during WeD (all *p* < 0.050). Males, females and adults performed more MVPA and TPA during WD than during WeD (all *p* < 0.050).

When comparing center time vs. outside-center time all participants spent more time in ST during center time (all *p* < 0.001). All participants performed greater amounts of LPA during center time than during outside-center time (all *p* < 0.050). Adults and females performed greater amounts of MVPA during center time than during outside-center time (*p* < 0.001; *p* = 0.001). On the other hand, males and older adults registered greater values of total PA during outside-center time than during center time (all *p* ≤ 0.050). When comparing steps per day, all participants performed more steps during center time than during outside-center time (all *p* < 0.010).

**Figure 1:**
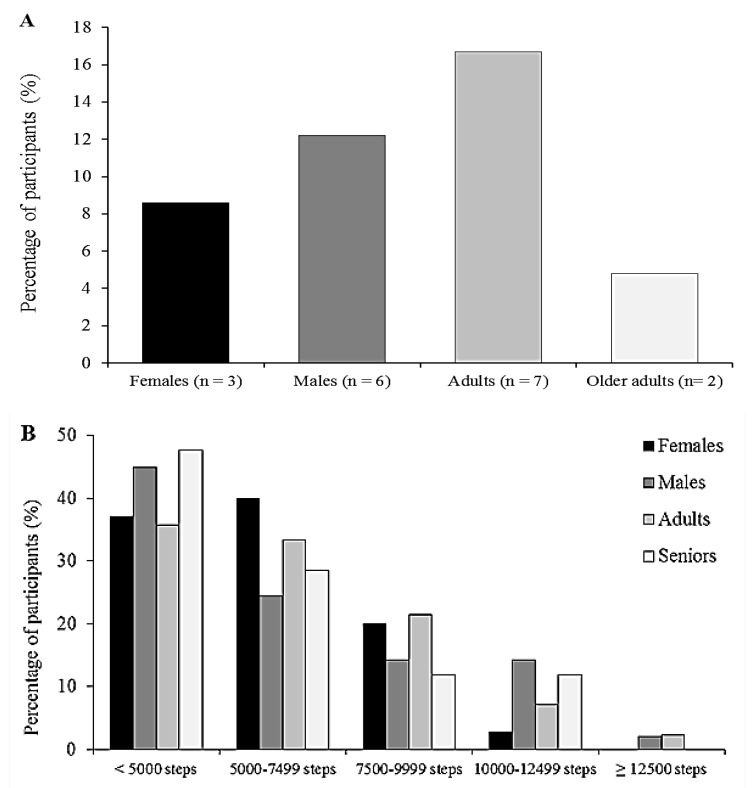
Percentage of participants who met the WHO recommendation for PA (**A**) and percentage of participants within daily step categories (**B**) by sex and age groups.

Figure 1 depicts the proportion of participants meeting WHO recommendations for PA (≥150 min per week of MVPA in 10-min bouts) and the percentage of participants within daily steps category. Overall, 9 (10.7%) participants met PA recommendations and 9 (10.7%) achieved 10,000 steps·day^−1^. When comparing by sex, 6 males (12.2%) and 3 females (8.6%) met the PA recommendation and 8 males (16.3%) and only 1 female (2.9%) achieved the recommended 10,000 steps·day^−1^, no significant differences were observed between sex. A total of 7 adults (16.7%) and 2 older adults (4.8%) met the PA recommendation while 4 adults (9.5%) and 5 older adults (11.9%) achieved the recommended 10,000 steps·day^−1^. No significant differences were observed among adults and older adults.

### 3.3. Sedentary Time Patterns

Table 4 presents the analysis for ST. In this study, the participants spent 79.4% of their waking time in ST. There were on average 65 sedentary bouts per day (lasting 6.0 ± 1.3 min) with on average 6 breaks per sedentary hour (lasting 6.7 ± 3.1 min). A significant difference was found for age groups where adults present a higher number of breaks per sedentary hour than older adults (*p* = 0.048) and the obese participants spent more time in ST than those participants with a normal BMI (*p* = 0.042). The number of bouts in sedentary behaviours per day was greater in participants classified as overweight and obese than in participants classified as normal weight (*p* ≤ 0.005). On average, the participants had 1 bout ≥30 min of ST per day, which accounted for 9.4% of ST, and only 0.3 bouts ≥60 min of ST per day, which accounted for 4.5% of ST (Table 5).

## 4. Discussion

The main finding of this study is that adults with ID perform more MVPA and TPA than older adults with ID, but no differences were found in ST. The results regarding MVPA and TPA are similar to those found in previous studies with general population and people with ID [31,32,33,46], where PA levels decrease with age. In addition, we found that adults with ID engaged in more MVPA and TPA than older adults with ID on WD and in center time. When the age group was divided by sex, it was found that the main differences in MVPA during WD and center time were due to the fact that adult males with ID perform more MVPA than older adults with ID. These results indicate that older males and females of the current study should be engaged in age and sex specific activities to increase their PA levels. Both sex and adults of the current study engaged in more MVPA, TPA and performed more steps during WD than during WeD. This is probably due to the fact that part of work tasks of the participants requires light to vigorous intensity physical labors such as cleaning and gardening.

Regarding the WHO recommendations on PA for health, ~17% of adults and ~5% of older adults in the present study achieved them. The value found for adults was higher than the value found by Dixon-Ibarra et al. [33] and similar to the values found in older adults with ID. When presented by sex group, ~12% of males and ~9% of females met the recommendation. Worryingly, in our study the percentage of people with ID achieving WHO recommendations was very low (~11%); nevertheless, this percentage is slightly higher than the percentage reported in the review by Dairo et al. [11] for people with ID (9%) and higher than the values found for US adults without ID where less than 5% of them were achieving the recommended amounts of PA for health [46]. On the other hand, the percentage of participants achieving the recommendations are lower than those reported in 2010 by WHO where around 23% of adults aged 18 and over were not active enough.

The average time for MVPA found in our study (~31 min·day^−1^) is higher than those presented by Melville et al. [8] (~13 min·day^−1^) and similar to those obtained by Phillips & Holland [32] (~36 min·day^−1^). However, when dividing the sample by age groups, adults averaged ~36 min of MVPA per day while older adults averaged ~25 min of MVPA per day. These values are higher than the results found by Dixon-Ibarra et al. [33] for adults and older adults with ID, which could be due to the different mean age of their groups, but are still lower than the values obtained in another study for a group of active adults without ID [21]. When splitting our data by sex, we found that the values of MVPA for males were lower than the values reported by Phillips et al. [32] and were similar for females. In the same line with another study [31] but in contrast with Phillips et al. [32], we found no differences for MVPA between sex. These differences between results could be explained by the age of the participants in the study by Phillips et al. [32], where the mean age was ~34 years old while in the current study the mean age was 44 years old. Also, the fact that 100% of participants in the current study were attending a day center might have contributed to increase the MVPA performed by them.

The values of LPA found in our study were similar to the values reported by Phillips et al. [32] and higher than the values reported by Melville et al. [8]. The difference with Melville et al. [8] could be due to the fact that their participants were 4 years older than the participants in this study and all of them presented a BMI ≥ 30 kg/m^2^.

Compared with another study assessing TPA in people with ID [32], we found that the TPA performed by the participants in this study was much lower. We hypothesised that this could be due to the older age of our participants and the lower amount of minutes spent in MVPA.

Overall, participants from this study accumulated more steps during WD and during center time. These differences could be due to different tasks that the participants performed at their work center (gardening, cleaning, tasks in the kitchen and cafeteria, etc.). During the WeD, adults had a higher step counts than older adults, which might be due to age-related decrease of physical functioning or to the lack of motivation or opportunities for older adults with ID to be part of leisure activities during the WeD [52].

The average number of steps per day reported in the review by Dairo et al. [11] (~6795 steps·day^−1^) is slightly higher than the average steps per day performed by our participants (6192 steps·day^−1^). Also, the number of daily steps in the current study are lower than daily steps reported by Hansen et al. [53] for people without ID (8038 steps·day^−1^). Moreover, in our study ~11% of the participants achieved ≥10,000 steps·day^−1^ while ~47% perform 5000 to 10,000 steps·day^−1^ and ~42% did not achieve 5000 steps·day^−1^. When comparing with other studies, we found that the proportion of adults with ID that accumulated the recommended quantity of steps per day in our study was lower than the values reported by other authors, who found that 14% to 45% of participants achieved the recommendation [29,30,54], but higher than the proportions reported by Dixon-Ibarra et al. [33]. Also, our study shows that a small proportion of participants (~27%) performed the mildest criterion of 7500 steps·day^−1^. This number of steps may maintain and promote health benefits to this specific population with ID [55].

When analyzing very sedentary population, such as people with ID, we hypothesize that the rule where only the accumulation of 150 min of moderate-intensity or 75 min of vigorous-intensity PA or some combination of the two per week in bouts of at least 10 min [4] will lead to health improvements may be very restrictive; and it might be possible that the accumulation of a volume of LPA and/or MVPA in bouts with a duration lower than 10 min might be less restrictive and really healthy PA [56]. Nevertheless, this hypothesis needs to be rigorously tested in future studies including people with ID.

The high level of sedentary activity and low adherence to PA recommendations found in our study are in concordance with the results found by several studies [8,32,33]. In general, adults and older adults from our study spent on average ~79% of their non-sleeping time as sedentary, while only ~17% and ~4% of the monitored time were spent in LPA and MVPA, respectively. The proportion of time spent in sedentary behaviour is higher than the results presented by another study [33] where people with ID spent ~61% of waking time in ST but in line with the review by Melville et al. [28] where ST range from 63% to 87.5%. In the current study, the percentage of non-sleeping time spent in ST was higher than the ~57% to ~61% of non-sleeping time spent in ST by people without ID [57,58]. It is worth keeping in mind that the choice of accelerometer cut-off points may affect the time spent in ST. We re-analyzed our data with the cut-off points proposed by Aguilar-Farías et al. [59] and we found that the participants of the current study spent ~66% of their non-sleeping time in ST.

When analyzing different periods of the week, we found that during accelerometer wearing time participants engaged in more ST during WD than WeD. In contrast to the results obtained by Dixon-Ibarra et al. (2013) [33] and independently of the period of the week (WD vs. WeD; center time vs. outside-center time), no differences between groups were found, which indicates that the problem of excessive time spent in ST is not dependent on the sex or age of the participants of the current study, but is a common behavioural pattern and, therefore, new strategies should be implemented in order to reduce this behaviour that is detrimental to their health.

Regarding the patterns of sedentary behaviour, ~86% of sedentary bouts occurred at bout durations of <10-min of which ~63% occurred at bout durations of <5-min and ~23% occurred at bout durations between 5-min and 10-min. In this study, we found less sedentary bouts per day than other authors, but the duration was greater than the reported by these authors [60]. The number of sedentary breaks in this study is lower than the sedentary breaks carried out by old people without ID [60,61], indicating that the risk of suffering metabolic disorders is higher in people with ID due to an excessive time spent in sedentary and lower quantities of sedentary breaks [58].

Sedentary behavior is ubiquitous in modern life and people with ID, who already suffer from lack of places and opportunities to include PA as part of their lifestyle, are not exempt of this harmful behaviour. The amount of time that adults with ID are inactive is problematic, especially if participants were pursuing sedentary activity during the non-wear time. Fortunately for sedentary and low active populations, reductions in mortality risk begin to accumulate with the first increase in PA beyond base-line [62], which means that even small increases in activity could provide substantial health benefits.

Possibilities to engage in PA programs may be hindered due to many social, cognitive, motor and behavioural factors associated with the presence of ID. Low participation in PA programs for adults with ID may be related to lack of appreciation of the benefits of PA, lack of support from their caregivers or difficulty in finding experienced personnel to train them [63]. Assessing the barriers that people with ID face to be part of structured PA programs is essential to later be able to provide them with the most suitable opportunities in accordance with their ID levels and necessities.

Taking into account the results of the study by Dunstan et al. [64] showing that even short bout of interruptions in sedentary time with light or moderate intensity walking significantly reduced the levels of postprandial glucose and insulin; we consider that focusing on replacing sedentary behaviour by light PA rather than on accumulating bouts ≥10-min of MVPA could be an achievable strategy to increase PA levels in people with ID.

A limitation of our study that should be highlighted is that PA may have been underestimated because ActiGraph accelerometers were mounted at the hip level and PA or movements with the upper-body were not measured. Also, these accelerometers cannot be worn during swimming or bathing activities. Cutoff points used in this study were developed for people without ID; therefore they might not be perfectly suitable to assess either PA or ST in population with ID. The cutoff points used to categorize PA intensity levels may underestimate energy expenditure and intensity in this population due to reduced coordination of movements, lower maximum oxygen consumption and increased mean weight [65,66]. In addition, the assigned work tasks in this specific center may be different to the general ID population; therefore, the levels of PA and ST of the participant of the current study might not be perfectly comparable with the rest of people with ID. Since multiple comparisons corrections were not applied in the present study in order to decrease the probability of getting false negative results due to the small size of the study, results of borderline significance need to be cautiously interpreted and will warrant confirmation in future studies. Finally, it is not possible to claim that our results are representative of the population of adults and older adults with ID because we have used a convenience sampling methodology to assessed PA levels and ST from one specific Spanish region.

## 5. Conclusions

This study provides novel and valuable detailed data on ST (number and duration of bouts, breaks and the total amount of ST) and PA patterns in a sample of adults and older adults with ID. It shows that PA levels of adults and older adults with ID involved in this study are very low and there is a high prevalence of sedentarism. The fact that only 10.7% of the participants met PA recommendations and/or achieved 10,000 steps·day^−1^ indicates that special attention should be paid to the creation of new strategies and well-designed and accessible health promotion programs for adults with ID, which must take into consideration the barriers that people with ID face to practice PA. These programs should focus not only on increasing PA levels but also on reducing sedentary levels.

On the other hand, future studies are needed to further investigate how PA levels and the excessive time spent in ST, bouts of particular duration and quantities of sedentary breaks are related to health outcomes and other risk factors of health in this unique population. These data and knowledge will help to target specific interventions to maintain their functional independence and provide opportunities for recreation and enjoyment that will help to improve their quality of life.

## Figures and Tables

**Table 1:** Descriptive characteristics of the participants.

Variables	All *n* = 92	Sex		*p*_1_	Age Groups		*p_2_*
		Males *n* = 53	Females *n* = 39		Adults *n* = 47	Older Adults *n* = 45	
Age (year)	44 (12)	45 (11)	43 (13)	0.576	35 (7)	54 (6)	**<0.001**
Height (cm)	160.5 (11.1)	165.2 (9.7)	154.1 (9.8)	**<0.001**	161.4 (12.3)	159.5 (9.5)	0.419
Weight (kg)	73.8 (14.3)	71.9 (111.7)	76.3 (17.1)	0.385	70.8 (14.0)	76.9 (14.2)	**0.044**
BMI (kg/m^2^)	28.9 (6.5)	26.4 (3.9)	32.2 (7.7)	**<0.001**	27.2 (4.4)	30.7 (7.7)	**0.009**
Normal (%)	26.1	37.7	10.3	**<0.001**	36.2	15.6	0.067
Overweight (%)	42.4	50.9	30.7		34.0	51.1	
Obese (%)	31.5	11.4	59.0		29.8	33.3	
WC (cm)	94.0 (15.7)	92.7 (11.8)	95.8 (19.7)	0.347	88.4 (12.1)	99.8 (16.9)	**<0.001**
Normal (%)	57.6	77.4	30.8	**<0.001**	66.0	48.9	0.098
Elevated (%)	42.4	22.6	69.2		34.0	51.1	
Body fat mass (%)	30.7 (10.0)	24.5 (7.4)	39.1 (6.2)	**<0.001**	30.4 (9.5)	31.2 (10.6)	0.706
Body fat-free mass (%)	32.8 (7.7)	37.2 (5.7)	26.9 (5.8)	**<0.001**	33.6 (7.1)	31.9 (8.3)	0.304

Note: data are expressed as mean (SD) or percentage of the group. *p*_1_ Difference between sex based on *t*-tests for continuous variables and chi-square for categorical variables. *p*_2_ Difference between age groups based on *t*-tests for continuous variables and chi-square for categorical variables. Statistically significant values are shown in bold (*p* ≤ 0.05). BMI, body mass index; WC, waist circumference.

**Table 2:** Patterns of sedentary time and physical activity throughout the week in adults and older adults with ID.

Variables	Average Day *n* = 84	Week Days *n* = 84	Weekend Days *n* = 79	*p*_1_	Center Time *n* = 84	Outside-Center Time *n* = 84	*p_2_*
Sedentary time (min·day^−1^)	612.9 (80.1)	624.3 (83.5)	583.6 (100.5)	**<0.001**	363.3 (36.1)	261.0 (67.4)	**<0.001**
Light PA (min·day^−1^)	128.3 (47.0)	133.0 (48.1)	117.6 (57.5)	**<0.001**	71.4 (30.3)	61.7 (26.0)	**<0.001**
MVPA (min·day^−1^)	30.8 (22.5)	32.7 (23.2)	26.8 (25.5)	**<0.001**	18.3 (13.4)	14.3 (13.9)	**0.001**
Total PA (counts·min^−1^)	251.9 (123.2)	258.9 (119.8)	236.0 (159.2)	**<0.001**	245.5 (119.0)	278.7 (166.8)	**0.048**
Steps (steps·day^−1^)	6192 (2814)	6523 (2807)	5378 (3686)	**<0.001**	3697 (1604)	2827 (1731)	**<0.001**
Wearing time (min·day^−1^) ^†^	772.0 (78.6)	790.0 (85.4)	728.0 (95.5)	**<0.001**	453.0 (37.0)	337.0 (76.0)	0.821

Note: the data presented above are raw means (SD). *p*_1_ Differences between week days vs. weekend days based on mixed models adjusted for differences in wearing time, sex and age group. *p_2_* Differences between center time vs. outside-center time based on mixed models adjusted for differences in wearing time, sex and age group. ^†^ Differences based on mixed models adjusted for sex and age group. Statistically significant values are shown in bold (*p* ≤ 0.05). PA, physical activity; MVPA, moderate to vigorous physical activity.

**Table 3:** Patterns of sedentary time and physical activity throughout the week in adults and older adults with ID by sex and age.

Variables	Sex		*p*-Values	Age Groups		*p*-Values
	Males *n* = 49 ^1^	Females *n* = 35 ^2^		Adults *n* = 42 ^3^	Older Adults *n* = 42 ^4^	
Average day ^†^						
Sedentary time (min·day^−1^)	607.7 (86.0)	620.2 (71.6)	0.369	599.7 (76.8)	626.0 (82.2)	0.155
Light PA (min·day^−1^)	130.5 (54.4)	125.2 (34.7)	0.922	121.8 (39.9)	134.8 (52.8)	0.247
MVPA (min·day^−1^)	32.1 (26.8)	29.0 (14.6)	0.913	36.3 (26.3)	25.3 (16.4)	**0.011**
Total PA (counts·min^−1^)	260.2 (146.4)	240.2 (80.8)	0.838	277.8 (138.8)	226.0 (100.4)	**0.038**
Steps (steps·day^−1^)	6389 (3313)	5916 (1923)	0.884	6539 (2960)	5844 (2650)	0.204
Weekdays ^†^						
Sedentary time (min·day^−1^)	623.1 (89.1)	625.9 (76.3)	0.641	608.4 (77.9)	640.2 (86.9)	0.396
Light PA (min·day^−1^)	132.5 (52.8)	133.8 (41.5)	0.512	127.8 (43.7)	138.2 (52.2)	0.502
MVPA (min·day^−1^)	33.6 (26.8)	31.4 (17.2)	0.874	39.3 (26.3)	26.0 (17.6)	**0.014**
Total PA (counts·min^−1^)	262.7 (137.3)	253.6 (91.6)	0.918	291.3 (129.9)	226.6 (100.3)	**0.042**
Steps (steps·day^−1^)	6622 (3222)	6386 (2133)	0.920	7047 (2970)	5999 (2564)	0.146
Weekend days ^†^						
Sedentary time (min·day^−1^)	568.3 (101.9)	606.1 (95.5)	0.069	586.6 (103.6)	580.7 (98.5)	0.942
Light PA (min·day^−1^)	125.8 (66.5)	105.6 (38.7)	0.260	108.6 (51.7)	126.4 (62.0)	0.067
MVPA (min·day^−1^)	29.0 (30.7)	23.5 (14.9)	0.746	28.4 (29.7)	25.2 (20.8)	0.822
Total PA (counts·min^−1^)	255.7 (189.9)	207.0 (93.7)	0.366	237.1 (180.3)	234.9 (137.8)	0.733
Steps (steps·day^−1^)	5787 (4286)	4777 (2509)	0.396	5211 (3573)	5540 (3831)	0.392
Center time ^†^						
Sedentary time (min·day^−1^)	363.8 (41.2)	362.5 (27.9)	0.976	362.6 (29.1)	363.9 (42.3)	0.853
Light PA (min·day^−1^)	70.3 (33.3)	72.9 (25.9)	0.388	69.7 (25.8)	73.1 (34.5)	0.300
MVPA (min·day^−1^)	18.0 (13.9)	18.8 (12.8)	0.469	22.6 (14.0)	14.0 (11.3)	**0.001**
Total PA (counts·min^−1^)	242.1 (125.5)	250.3 (110.8)	0.558	277.6 (119.9)	213.4 (110.3)	**0.017**
Steps (steps·day^−1^)	3654 (1714)	3756 (1457)	0.617	4121 (1517)	3273 (1593)	**0.047**
Outside-center time ^†^						
Sedentary time (min·day^−1^)	259.3 (70.7)	263.4 (63.3)	0.231	245.8 (68.7)	276.2 (63.3)	0.473
Light PA (min·day^−1^)	62.1 (25.9)	60.9 (26.5)	0.992	58.1 (28.5)	65.1 (23.0)	0.242
MVPA (min·day^−1^)	15.6 (16.5)	12.6 (9.1)	0.409	16.6 (17.9)	12.0 (7.8)	0.066
Total PA (counts·min^−1^)	293.9 (197.5)	257.5 (109.6)	0.441	315.2 (206.0)	242.3 (105.6)	0.135
Steps (steps·day^−1^)	2967 (1970)	2630 (1328)	0.492	2927 (2075)	2726 (1319)	0.550

Note: the data presented above are raw means (SD). ^1^ *n* = 47 for weekend days. ^2^ *n* = 32 for weekend days. ^3^ *n* = 39 for weekend days. ^4^ *n* = 40 for weekend days. ^†^ Differences between sex based on mixed models adjusted for age group; difference between age groups based on mixed models adjusted for sex. Models were adjusted for differences in wearing time. Statistically significant values are shown in bold (*p* ≤ 0.05). PA, physical activity; MVPA, moderate to vigorous physical activity.

**Table 4:** Time in sedentary, percentage of sedentary behavior·day^−1^, number of sedentary bouts·day^−1^, and number of breaks per sedentary hour.

Variables	Sedentary Behavior (min·day^−1^)	*p*_1_	Percentage of Wear Time in Sedentary Behavior·day^−1^	*p*_2_	Number of Bouts in Sedentary Behavior·day^−1^	*p*_3_	Number of Breaks per Sedentary Hour	*p*_4_
All participants	612.9 (80.1)		79.4 (6.5)		64.8 (11.7)		6.2 (0.7)	
Sex ^†^		0.369		0.431		0.491		0.593
Males	607.7 (86.0)		78.9 (7.4)		64.1 (12.1)		6.3 (0.6)	
Females	620.2 (71.6)		80.1 (4.9)		65.8 (11.4)		6.2 (0.7)	
Age group ^†^		0.155		0.654		0.553		**0.048**
Adults	599.7 (76.8)		79.1 (5.6)		64.1 (9.5)		6.4 (0.6)	
Older Adults	626.0 (82.2)		79.7 (7.3)		65.5 (13.7)		6.1 (0.7)	
BMI (kg/m^2^)		**0.039**		0.111		**0.005**		0.138
Normal	577.2 (90.8)		77.0 (8.1)		57.9 (11.1)		6.5 (0.6)	
Overweight	619.4 (74.1)		79.9 (5.5)		66.7 (10.4) ^§^		6.2 (0.6)	
Obese	634.0 (71.1) ^§^		80.7 (5.8)		68.0 (11.9) ^§^		6.1 (0.7)	

Note: data are expressed as mean (SD). *p*_1_ Between age groups, sex, BMI category difference in sedentary behavior (min·day^−1^). *p*_2_ Between age groups, sex, BMI category difference in sedentary behavior·day^−1^ (%). *p*_3_ Between age groups, sex, BMI category difference in the number of bouts in sedentary behavior·day^−1^. *p*_4_ Between age groups, sex, BMI category difference in the number of breaks per sedentary hour. ^†^ Differences between age groups based on linear models adjusted for sex; differences between sex based on linear models adjusted for age group. ^§^ Significant difference (*p* ≤ 0.05) when compared to Normal BMI. Statistically significant values are shown in bold (*p* ≤ 0.05). BMI, body mass index.

**Table 5:** Number of sedentary bouts·day^−1^, percentage of sedentary bouts and percentage of sedentary time of various bouts duration.

Bout Duration (min)	Number of Sedentary Bouts per day ^†^	Percentage of Sedentary Bouts of *n* min ^§^	Percentage of Sedentary Time in Bouts of *n* min *
2+	64.8 (11.7)	100	100
5+	23.8 (7.0)	36.3 (8.6)	65.8 (11.2)
10+	9.0 (3.9)	13.8 (5.7)	39.7 (12.5)
20+	2.6 (1.6)	4.0 (2.7)	18.6 (9.9)
30+	1.0 (0.8)	1.6 (1.4)	9.4 (6.8)
40+	0.6 (0.4)	0.9 (0.8)	6.6 (4.8)
50+	0.4 (0.3)	0.6 (0.5)	5.1 (3.5)
60+	0.3 (0.2)	0.5 (0.3)	4.5 (2.4)

Note: data are expressed as mean (SD). ^†^ A bout of sedentary behavior is defined as a period of consecutive minutes where the accelerometer register <100 counts·min^−1^. ^§^ Number of sedentary bouts of *n* min/number of sedentary bouts of 2 min. * Length of sedentary bouts of *n* min/length of sedentary bouts of 2 min.
